# An Ultraflexible Electrode Array for Large‐Scale Chronic Recording in the Nonhuman Primate Brain

**DOI:** 10.1002/advs.202302333

**Published:** 2023-10-23

**Authors:** Yixin Tian, Jiapeng Yin, Chengyao Wang, Zhenliang He, Jingyi Xie, Xiaoshan Feng, Yang Zhou, Tianyu Ma, Yang Xie, Xue Li, Tianming Yang, Chi Ren, Chengyu Li, Zhengtuo Zhao

**Affiliations:** ^1^ Institute of Neuroscience Center for Excellence in Brain Science and Intelligence Technology Chinese Academy of Sciences Shanghai 200031 China; ^2^ Shanghai Center for Brain Science and Brain‐Inspired Technology Shanghai 201602 China; ^3^ Lingang Laboratory Shanghai 200031 China; ^4^ Institute of Neuroscience State Key Laboratory of Neuroscience, Center for Excellence in Brain Science and Intelligence Technology Chinese Academy of Sciences Shanghai 200031 China; ^5^ University of Chinese Academy of Sciences Beijing 100049 China; ^6^ Institute of Neuroscience Key Laboratory of Primate Neurobiology, Center for Excellence in Brain Science and Intelligence Technology Chinese Academy of Sciences Shanghai 200031 China

**Keywords:** brain–machine interfaces, motor cortex, nonhuman primates, single‐unit recordings, ultraflexible electrode arrays, visual cortex

## Abstract

Single‐unit (SU) recording in nonhuman primates (NHPs) is indispensible in the quest of how the brain works, yet electrodes currently used for the NHP brain are limited in signal longevity, stability, and spatial coverage. Using new structural materials, microfabrication, and penetration techniques, we develop a mechanically robust ultraflexible, 1 µm thin electrode array (MERF) that enables pial penetration and high‐density, large‐scale, and chronic recording of neurons along both vertical and horizontal cortical axes in the nonhuman primate brain. Recording from three monkeys yields 2,913 SUs from 1,065 functional recording channels (up to 240 days), with some SUs tracked for up to 2 months. Recording from the primary visual cortex (V1) reveals that neurons with similar orientation preferences for visual stimuli exhibited higher spike correlation. Furthermore, simultaneously recorded neurons in different cortical layers of the primary motor cortex (M1) show preferential firing for hand movements of different directions. Finally, it is shown that a linear decoder trained with neuronal spiking activity across M1 layers during monkey's hand movements can be used to achieve on‐line control of cursor movement. Thus, the MERF electrode array offers a new tool for basic neuroscience studies and brain–machine interface (BMI) applications in the primate brain.

## Graphical abstract

A mechanically robust ultraflexible, 1 µm thin electrode array (MERF) designed for single‐unit recording in nonhuman primates is presented. The MERF array enables pial penetration and high‐density, large‐scale, and chronic recording of neurons along both vertical and horizontal cortical axes in the nonhuman primate brain, offering a new tool for basic neuroscience studies and brain–machine interface applications.

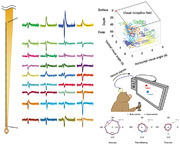

## 1 Introduction

Understanding the dynamics of neural circuit activity underlying various brain functions requires long‐term monitoring of neuronal spiking over large populations of neurons with high spatial and temporal resolution.^[^ ^1^, ^2^, ^3^ ^]^ Existing implanted electrodes, including those capable of nonsuperficial recording of SU activity with sub‐millisecond resolution, have greatly contributed to both basic and applied neuroscience.^[^ ^4^, ^5^, ^6^, ^7^, ^8^, ^9^, ^10^, ^11^ ^]^ Conventional electrodes are mainly metal wire‐based arrays or silicon‐based probes fabricated with MEMS (Micro‐Electro‐Mechanical System) technology.^[^ ^12^, ^13^, ^14^, ^15^, ^16^ ^]^ Long‐term implantation of these rigid probes may cause damage to the surrounding brain tissues due to the relative motion between the rigid probe and the soft tissue, especially in large awake animals. This could result in tissue reactions, such as chronic inflammation and glial scar formation, leading to the deterioration of recording quality.^[^ ^17^, ^18^, ^19^ ^]^ Recently, flexible cellular‐scale microelectrodes have shown promise in resolving these problems.^[^ ^20^ ^]^ These electrodes have excellent tissue compatibility and minimal invasiveness that allow the stable recording of single‐neuron activity for up to 10 months in rodents.^[^ ^21^ ^]^ However, the longevity and stability of recording by such flexible probes have yet to be achieved in large animals, especially in NHPs.^[^ ^22^ ^]^

Because of their evolutionary proximity to humans, NHPs play an irreplaceable role in studying higher cognitive functions and brain diseases.^[^ ^23^, ^24^ ^]^ However, there are significant challenges to stable and long‐term electrophysiological recording from NHPs. First, the thick and tough meninges covering the primate brain are difficult to penetrate by microelectrodes. Second, large tissue displacement relative to electrodes during surgery and postsurgical recording may elicit exaggerated immune response and reduce recording stability.^[^ ^25^ ^]^ Third, the large brain volume of NHPs posts difficulties for recording from deep brain structures. Most recordings in NHPs previously reported were achieved by using rigid electrodes. Among these electrodes, metal microwire electrodes are usually accompanied with a microdrive system for positioning, but it is difficult to track SUs chronically.^[^ ^12^ ^]^ The widely used “Utah” array achieved long‐term recording at sparsely distributed locations (at 400 µm spacing) on a horizontal plane of the NHP and human cortex, yielding insights into brain functions and applications for BMIs.^[^ ^26^, ^27^ ^]^ Notably, this method is restricted to superficial cortical layers, and known to show some signal instability during long‐term recording due to local immune reactions.^[^ ^28^, ^29^ ^]^ Other linear‐array silicon electrodes have recently been reported for recording in NHP brains. For example, “Neuropixels” probes have achieved acute high‐density recording through hundreds of channels spanning the cortex and deep brain structures of awake behaving monkeys.^[^ ^30^, ^31^, ^32^ ^]^ Efforts to implant silicon‐based probes chronically in NHPs have been made by applying new fabrication and assembly strategies, such as using a thicker single‐shank probe mounted with a skull‐fixed microdrive system,^[^ ^33^ ^]^ or stacking customized modules to form versatile electrode arrays.^[^ ^34^ ^]^ However, the long‐term stability of silicon‐based probes is still limited due to their rigid structure relative to the soft brain tissue. On the other hand, the flexible polymeric neural probes in NHPs have been either primarily used on peripheral nerves, such as radial nerves,^[^ ^35^ ^]^ or functioned in a similar way as SEEG electrodes.^[^ ^36^, ^37^ ^]^ Although a recent study has achieved 1 year recordings from the visual cortex of NHPs using polymeric neural probes, the recording scale and quality were unsatisfactory, with only 125 electrodes implanted in a single monkey.^[^ ^38^ ^]^ Overall, large‐scale, long‐term, and volumetric recordings from the NHP brain remain a challenge in the field.

In this study, we successfully performed long‐term, large‐scale, and stable recordings of SU activity in the NHP brain, using a newly developed mechanically robust ultraflexible electrode array (termed “MERF”). We introduced new structural materials and an electrode design that increased the mechanical strength of the electrodes while maintaining the subcellular dimension and ultraflexibility of the electrodes. This allowed the electrode penetration through the arachnoid and pia matter of the NHP brain, and provided mechanical compatibility of the electrode with the brain tissue. We implanted MERF electrode arrays with hundreds of recording channels into the V1 and M1 of two cynomolgus monkeys and one rhesus monkey. SU activity was recorded during the period of over 8 months in which some SUs were continuously tracked for up to 2 months. To demonstrate the quality of recording, we characterized the orientation tuning and the spike correlation of the V1 neurons as well as the hand movement direction selectivity of the M1 neurons. We further trained a linear decoder with M1 neuronal spiking activity associated with the monkey's hand movements to control a cursor on a computer screen, and achieved the on‐line control of the cursor trajectory based on the recorded neural activity of the M1 neurons. Together, our results demonstrated the capability of this new ultraflexible microelectrode for stable, large‐scale long‐term recordings of single‐neuron activity in NHPs.

## 2 Results

### 2.1 Construction and Characterization of the MERF Electrode Array

**Figure 1:**
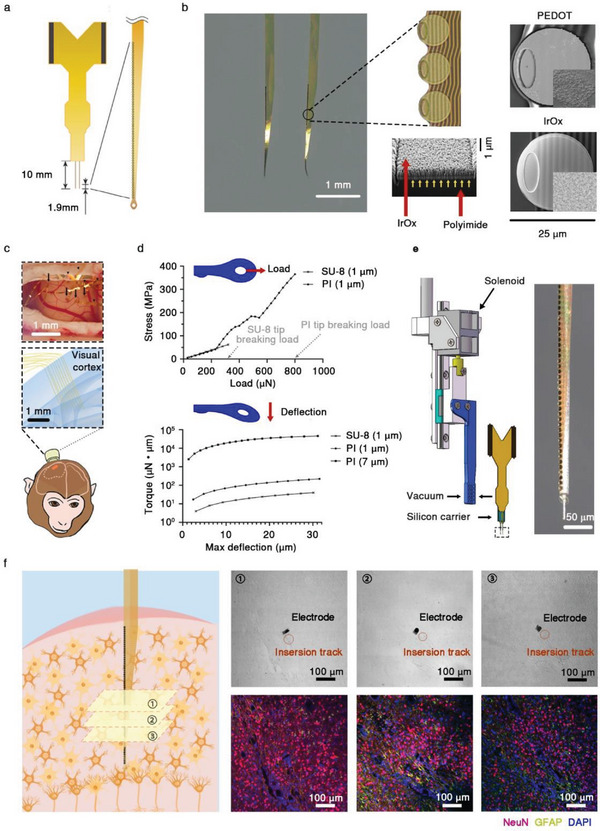
The design of the MERF electrode array. a) Schematic diagram showing an example MERF electrode array containing 2 shanks. Each shank of this design contains one lane of 64 recording sites evenly distributed along the flexible PI shank. b) The image of a MERF electrode array and electrode design. Left, an image of MERF shank showing an electrode array. The circular electrodes are shown at a higher resolution, with top and side views. Connecting wires are marked by yellow arrows. Right, the scanning EM images of two circular electrodes with two different coatings (IrOx and PEDOT). Insets, electrode surface at a higher resolution. c) MERF implantation in the monkey brain. Top, image of open dura window showing the cortical surface of monkey after MERF implantation. Arrows, MERF insertion sites; arrowheads, external connecting wires. Bottom, schematic diagram illustrating the cortical locations of implanted multiple MERF arrays into the monkey V1. d) FEA simulating the stretching (top) and bending force (bottom) exerted on a model shank tip. Top, 1 µm thick PI‐made shank withstands greater stretching load than that of SU‐8‐made shank of the same size, as shown by the higher breaking load. Bottom, data depict torque forces required to produce various lengths of maximal deflection, for MERF shank (PI‐made, 1 µm), NET shank (SU‐8‐made, 1‐µm), and Neurolink shank (PI‐made, 7 µm). e) Schematic diagram of the micromanipulator‐controlled implantation device for inserting MERF into the brain tissue. The MERF array with its flexible connector (the yellow part) was adhered to the implantation device by vacuum suction, and the solenoid drove the rapid injection of electrodes via a slider and a track. f) Microscopic images of consecutive tissue sections around the implanted MERF array at 3 months after implantation. Left, schematic diagram showing three consecutive sections centered around the electrode array in the monkey brain. Right, bright‐field microscopic images showing a piece of shank remained embedded in the brain tissue, with the insertion track (red circle) after needle removal. Immunostaining images of the three sections for the glial marker GFAP (green) and the neuron marker NeuN (red), together with DAPI (blue) staining of cell nuclei, showing no substantial difference in cellular organization between the tissues around the implanted electrode and at a distance. Scare bar = 100 µm.

To perform large‐scale, long‐term, and volumetric recordings of SU activity from the NHP brain, we designed a new type of mechanical robust ultraflexible electrode array (termed “MERF”) with cellular dimension. Each MERF comprised 2 shanks and 64 channels on each shank (**Figure** **1**a). For interconnects, the minimal line width, spacing of interconnects, and the line resistance were 1.5, 1.5 µm, and 160 Ohm mm^−1^, respectively. The electrode contacts were placed at the edge of each shank for proximity to nearby neurons. The effective recording length of the shank was customized to be 1.9 mm to ensure full‐depth coverage of the NHP cortex. The contact pad of each electrode had a diameter of 25 µm and was coated with IrOx (iridium oxide) or PEDOT:PSS [poly(3,4‐ethylenedioxythiophene) polystyrene sulfonate:poly(styrene sulfonate] to reduce the impedance to ≈100 kΩ for a better signal‐to‐noise ratio (SNR) (Figure 1b). The modular design of MERF enabled the scalability of the spatial coverage for volume recordings and the flexibility in the spatial arrangement for electrode locations (Figure 1c).

To ensure both the ultraflexibility for stable long‐term recordings and the robust mechanical strength for the pial penetration during MERF implantation, we selected polyimide (PI) as the base material of the electrodes rather than SU‐8, which is the negative photoresist previously used as the insulation material for the ultraflexible electrode in rodents,^[^ ^17^, ^21^ ^]^ since we found that SU‐8 probes of the same thickness as that used in rodents failed to penetrate the monkey pia matter.^[^ ^39^ ^]^ We were able to fabricate the entire electrode shank with PI as the base material down to a thickness of 1 µm. At this thickness, PI provided ≈5.8 fold higher tensile strength than that of SU‐8 (Figure 1d, top), favorable for the pia penetration and the electrode withdrawal after recordings. Notably, compared with previous flexible PI shanks (with ≈7 µm thickness),^[^ ^18^ ^]^ the marked reduction in the shank thickness of MERF resulted in more than two orders of magnitude of improvement in structural flexibility, based on the theoretical estimate and the finite‐element analysis (FEA, Figure 1d, bottom; and Movie S1, Supporting Information). The electrode size was reduced by several orders of magnitude as compared to other recording electrodes for primate brains (Table S1 and Figure S1, Supporting Information). The cross‐sectional area of a MERF shank was only 100 µm^2^, comparable to that of neuronal soma. The unique mechanical compliance and miniature footprint ensured improved biocompatibility, as indicated by immunohistology results (Figure 1f). We did not observe significant glial activation or decreased neuronal density around the insertion site compared to distant tissues 3 months postimplantation, suggesting minimal tissue damage and immune reactions induced by MERF array implantation. Compared to silicon‐based probes, the cross‐sectional area of MERF arrays is significantly smaller (Table S1, Supporting Information). In contrast to a higher degree of tissue encapsulation and reduced neuronal density after long‐term implantation of rigid probes (e.g., “Utah” arrays),^[^ ^19^ ^]^ the tissue‐friendly properties of MERF arrays are favorable for long‐term recording. In addition, to examine the long‐term stability of our PI‐made MERF arrays, we performed accelerated aging tests (maintained at a temperature of 60 °C in 1 × PBS), in which the in vitro test duration is equal to 5 times of the duration in vivo.^[^ ^40^ ^]^ The impedance remained stable throughout the 46‐day accelerated aging test (equal to 230 days in vivo, *p* = 0.986, *n* = 31 electrode sites, ANOVA test, Figure S2a, Supporting Information), suggesting a good stability of the MERF arrays for long‐term implantation.

To facilitate penetrations of the tough arachnoid and pia maters of NHPs, we developed a high‐speed insertion method. In a typical implantation, the MERF shank was mechanically coupled to the tip of a tungsten shuttle wire using a needle‐and‐thread mechanism^[^ ^17^ ^]^ and delivered into the cortical tissue at a speed of 1.25 m s^−1^, driven by a solenoid (Figure 1e). In vitro test in 0.5% agarose gel showed that removing the shuttle wires only caused tiny retraction of MERF array shanks (mean retraction distance <80 µm, *n* = 7 shanks, Figure S2b, Supporting Information). A customized chamber was used to protect the electrode array after the implantation for long‐term recordings (Figure S3a,b, Supporting Information). We implemented a lightweight backend connection by using customized flexible printed circuits for electrophysiological recordings, together with a scalable neural data acquisition system (Figure S3c, Supporting Information). The performance of MERF in large‐scale chronic recordings in behaving monkeys is described as follows.

### 2.2 Long‐Term Recordings by MERF in Behaving Macaque Monkeys

**Figure 2:**
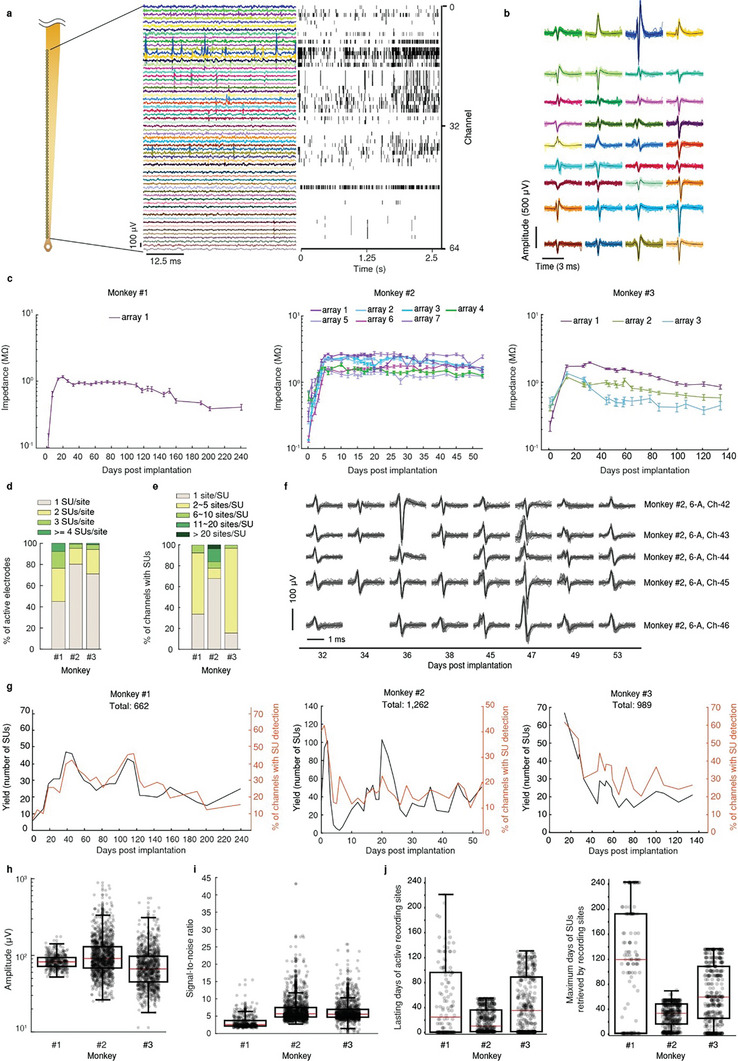
Long‐term recordings by MERF in awake monkeys. a) Sample voltage traces (300–6000 Hz band‐pass filtered, over 50 ms) and raster plots of the SU firing (2.5 s), recorded from one shank (64 electrodes) in V1 of monkey #2, at 53 days postimplantation. b) Waveforms of all putative SUs isolated at 53 days postimplantation, with each SU represented by the average of 50 traces. c) The averaged impedance of all functional electrodes over time (*n* = 115, 633, and 317 functional electrodes from 2, 14, and 6 shanks in monkey #1, 2, and 3, respectively, mean ± SEM). d) The number of SUs recorded per active electrode, for 3 monkeys. e) The average number of electrodes detecting the same SU in 3 monkeys. f) Examples of SU waveforms detected by a continuous row of multiple active electrodes in several recording sessions, indicating that an SU was detected by multiple adjacent channels. Each waveform represented by the average of 50 traces. g) Total number of SUs and percentage of active electrodes over time, for all MERF arrays implanted in the three monkeys (*n* = 662, 1262, 989 SUs in monkey #1, 2, and 3, respectively). h) Peak‐to‐trough amplitude of the averaged waveforms of all SUs from three monkeys (*n* = 662, 1262, 989 SUs in monkey #1, 2, and 3, respectively). i) SNR of all SUs (*n* = 662, 1262, 989 SUs in monkey #1, 2, and 3, respectively). j) Long‐term recording properties of the MERF array. Left, number of days for recording sites as continuously active sites. Right, the last day as active site for a given channel post implantation. Boxed plots in (h), (i), and (j): red lines indicate median, bottom and top box edges indicate percentiles of 25% and 75%, respectively.

We have evaluated the long‐term recording performance of MERF in three macaques (Table S2, Supporting Information). We implanted one electrode array in monkey #1 (in M1), seven in monkey #2 (in V1), and three in monkey #3 (in V1), successfully obtained a total of 115, 633, and 317 functional channels (electrode impedance <4 MΩ), respectively, in each monkey. Out of the total electrodes, the percentage of functional channels in the three monkeys was 89.8%, 70.6%, and 82.6%, respectively. Electrophysiological recordings by these MERF arrays yielded spike signals with high SNR in many channels (**Figure** **2**a,b). The average impedance per electrode progressively increased during the first 2 weeks after implantation, and became stabilized afterward for long periods (up to eight months) in all three monkeys (Figure 2c). For all functional electrodes, the average impedances during stable periods in the three monkeys were 0.82 ± 0.01 MΩ, 1.50 ± 0.01 MΩ, and 0.91 ± 0.01 MΩ, respectively. The average increase of impedance in the three monkeys was 0.35 ± 0.046 MΩ, 1.11 ± 0.043 MΩ, and 0.50 ± 0.054 MΩ, respectively. We identified SUs in our recordings that represented activity of single neurons using the automatic spike‐sorting toolbox Mountainsort^[^ ^41^ ^]^ or Kilosort^[^ ^42^ ^]^ with stringent criteria and curation procedures (Experimental Section). We defined functional channels that could resolve SU activity as active channels, and found that 34.6% ± 18.4% (*n* = 3 monkeys, mean ± SEM) of the active channels could record multiple neurons (Figure 2d) and 60.9% ± 26.3% of the neurons were recorded from multiple active channels (Figure 2e,f), facilitating the isolation of SUs (Figure S4, Supporting Information).

After an initial variation following MERF implantation, the SU yields and percentage of active channels became stable at a level (Figure 2g). For the three monkeys, the average amplitude of SUs and SNR of recording were 85, 113, 80 µV and 3, 7, 6, respectively (Figure 2h,i). The SNR from monkey #1 was relatively low due to extra noise induced by the monkey's hand movements when performing a motor task during the recording. The long‐term recoding performance was summarized by the average duration (49, 19, and 45 days in monkey #1, #2, and #3, respectively) for a given channel to record spikes in successive recording sessions (Figure 2j, left). In these experiments, the longest duration for a channel to resolve SUs (the last session that this channel was able to resolve spikes since electrode implantation), was 112, 30, and 62 days in monkey #1, #2, and #3, respectively (Figure 2j, right).

In total, we obtained 662 high‐quality SUs from 25 recording sessions in monkey #1, 1262 SUs from 31 sessions in monkey #2, and 989 SUs from 52 sessions in monkey #3. Besides SUs, we also recorded the multiunit events, which represent composite spiking activity of small groups of neighboring neurons. These results demonstrate that MERF is capable of long‐term recording in NHPs, despite variability in surgical and electrode implantation procedures among different laboratories (Table S2, Supporting Information).

### 2.3 MERF Revealed Functional Features of V1 Neurons

**Figure 3:**
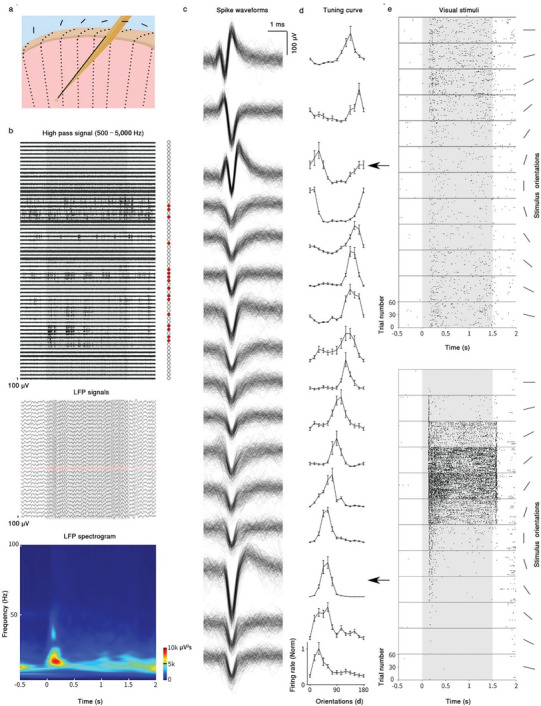
MERF array revealed functional features of V1 neurons. a) Schematic diagram showing the oblique penetration of the MERF shank relative to the vertical axis of the cortex, with the linear array of electrodes spanning several adjacent orientation columns of V1. b) An example recording trial in response to sinewave grating visual stimulus at 46 days postimplantation. Top, high pass filtered signals (500–5000 Hz) from 64 channels on the same shank, relative channel locations indicated as circles to the right. Middle, LFP signals (0.5–300 Hz) from bottom 32 channels. Bottom, LFP spectrogram from one example channel (in red of the above LFP panel). The shaded areas indicate the application of visual stimulus. c) Spike waveforms of example SUs in (b) (red filled channels). One hundred waveforms are shown for each channel with the same scale for comparison. d) Orientation tuning curves of SUs (as in (c)) in response to sinewave grating (mean ± SEM). Firing rates were normalized by that of the most active orientation for each unit. e) Raster plots of two example SUs in (c) as indicated by arrows. The bars to the right show the stimulus orientations. The shaded areas indicate the visual‐stimulus application.

An important feature of MERF is the multiple linear array of recording sites that are capable of capturing neuronal activity at different depths of the cortex. We have implanted the MERF arrays into the V1 and M1 of awake macaque monkeys to evaluate this capability of MERF. For monkey #2, we implanted MERF arrays in V1 and recorded neural activity during the exposure to visual stimuli of drifting gratings at various angles (**Figure** **3**a). An example recording obtained at 46 days postimplantation showed stimulus‐related local field potential (LFP) activity (<300 Hz) throughout the entire recording shank, and a clear stimulus onset‐evoked activity at beta and low‐gamma range (Figure 3b, middle and bottom). Importantly, we observed SU spiking activity at many active channels (filtered at >1 kHz) (Figure 3b, top and 3c). We further quantified the visual responses by measuring the tunning properties of various SUs to grating stimuli of different angles and found that 63% of the recorded V1 neurons showed clear orientation selectivity, with orientation selectivity index (OSI) > 0.3 (Figure 3d,e). The sharp tuning curves of these orientation‐selective neurons validated the recording quality of MERF. We found that SUs recorded from nearby channels along the same shank exhibited similar preferred orientations, which is consistent with the columnar microarchitecture revealed by prior studies.^[^ ^43^ ^]^ Notably, in the example of recording shown in Figure 3d, there was a progressive shift of preferred orientation of SUs along the shank, as a result of oblique penetration of the MERF shank relative to the vertical axis of the cortex, with the linear array of electrodes spanning several adjacent orientation columns of the V1 (Figure 3a).

**Figure 4:**
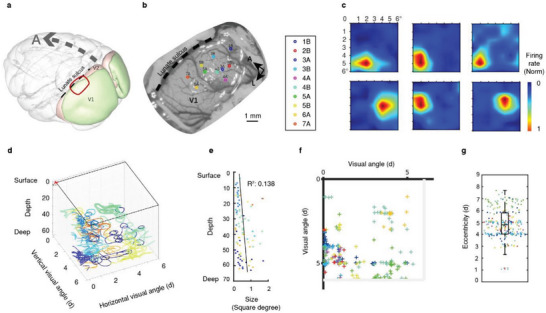
RF mapping across V1 through simultaneous multisite recording. a) Schematic diagram showing the durotomy area (red circle) of monkey #2. The transparent 3D model of the brain was from scalablebrainatlas.incf.org.^[^ ^63^, ^64^ ^]^ b) Implantation sites (circles) relative to the cortex. Number indicates ID of MERF array and A/B represents shank ID of an array. c) Heatmaps showing RFs of SUs of an example recording session in response to visual stimuli (circle of drifting grating) at different locations. Fixation point locates at coordinate (0,0). d) RFs of SUs in different cortical depth reconstructed in 3D space. e) RF size in relation to cortical depth. Noted the larger RF in deeper cortical tissue (*p* = 0.029, mixed‐effects model). f,g) The distribution of RF eccentricities of SUs for this session. Color code in (d)–(g) depicts the relative shanks as in (b).

Sequential implantation of MERF arrays allowed us to perform multisite recording across a cortical region. As shown by the recordings from V1 of monkey #2 (**Figure** **4**a,b), we were able to implant 10 MERF array shanks via 7 separate insertions, covering a cortical volume of about 4 × 6 × 3 mm^3^ with 640 recording sites. Receptive fields (RFs) of all SUs recorded from different layers were mapped simultaneously with drifting sinusoidal grating patches flashing at different locations in the visual field (Figure 4c) and visualized in 3D within the recorded cortical volume (Figure 4d; and Movie S2, Supporting Information). Such high‐throughput volumetric recordings of neuronal activity could greatly facilitates the study of neural dynamics within the cortical volume. Overall, the average RF size for the recorded neurons was less than 2° and the RF size increased gradually with the cortical depth of the recording electrode (*p* = 0.029, mixed‐effects model, Figure 4e), consistent with previous findings.^[^ ^44^, ^45^ ^]^ As the craniotomy was performed several centimeters away from the foveal area, the eccentricity of RFs was restricted to 5 ± 2° (mean ± SEM, Figure 4f,g). These results suggest that the MERF array enables us to study the functional properties of cortical neurons with a high resolution and efficiency over a large cortical volume.

### 2.4 On‐Line Motor Control with MERF‐Recorded M1 Signals

**Figure 5:**
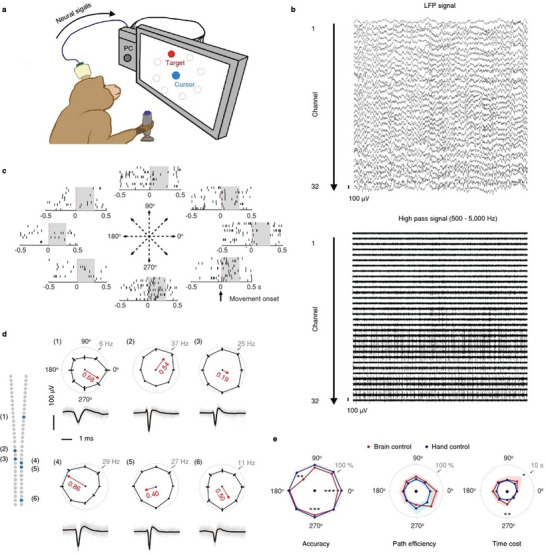
On‐line motor control with MERF‐recorded M1 signals in monkey #1. a) Schematic diagram showing the center‐out task in monkey #1. In some trials, the joystick was disconnected and the cursor was controlled by the neural activity recorded through a MERF array. b) Neuronal activity recoded from one MERF shank in performing the task. Top, LFP signals (0.5–300 Hz). Bottom, high pass filtered signals (500–5000 Hz). c) Raster plot of one example SU showing different firing rates toward different target directions. The shaded area indicate the epoch (0–300 ms relative to the movement onset) used to calculate directional tuning. d) Directional tuning of 6 representative SUs recorded along the same shank. Each panel shows the firing rate (mean ± SEM) and averaged waveform of each example unit during the center‐out task. The direction of the red tick indicates the preferred direction of each SU, and the length indicates the preference strength. Shank locations of 6 SUs are shown as filled blue circles in the panel to the left. e) Accuracy (left), path efficiency (middle), and time cost (right) for brain and hand controlled cursors. For (left), two‐tailed bootstrap test, 2000 times, corrected for multiple comparisons by false discovery rate, colored lines indicate the median, shading indicate the percentiles of 25% and 75% (Red, brain control; Blue, hand control). For (middle) and (right), Mann–Whitney U test, corrected for multiple comparisons by false discovery rate. *p < 0.05, **p < 0.01, ***p < 0.001.

In a separate set of experiments, we implanted a MERF electrode array into the M1 of monkey #1 after it was trained to hand‐manipulate a joystick to move a cursor on a computer screen to reach a target (**Figure** **5**a,b). Consistent with previous findings,^[^ ^46^, ^47^ ^]^ SUs recorded at different cortical depths along the same shank in M1 showed preferences of joystick movement directions (Figure 5c,d). To evaluate whether neural signals recorded by MERF contained sufficient information for us to decode cursor trajectories, we disconnected the joystick from the system and used the recorded M1 signals to directly control the cursor with a pretrained linear decoder (Movie S3, Supporting Information).

We compared the performance of the brain‐signal control system against the monkey's hand control using three evaluation metrics: accuracy, time cost, and path efficiency. We found no significant differences in time cost and path efficiency in most target directions between two types of control systems and only a slightly lower accuracy in the brain‐signal control system (Figure 5e). Thus, we demonstrated that neural signals recorded from various cortical layers by the MERF electrode array could provide enough information for on‐line 2D motor control.

### 2.5 Functional Coupling and Long‐Term Stability of Spiking Activity Revealed by MERF

**Figure 6:**
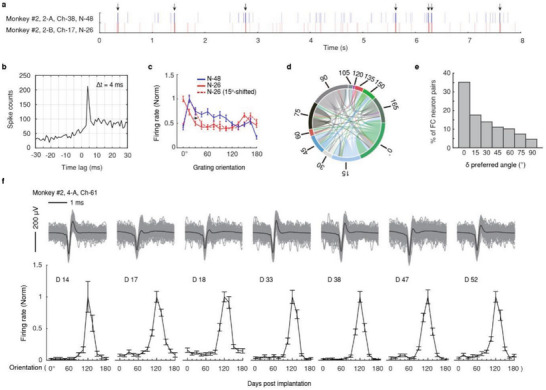
FC and long‐term stability of spiking activity revealed by MERF. a) Raster plot of one trials for an example functionally coupled neuronal pair in monkey #2. Arrows indicate functionally coupled spiking events (directional and correlated firing within 10 ms). b) Spike correlogram of the example neuronal pair in (a), showing spike counts of the following neuron N‐26 after spiking of the leading neuron N‐48. c) Turning curve of the two neurons in (a),(b) in response to visual stimuli (sinewave grating). Noted that the preferred orientation of N‐26 was close to that of N‐48. d) The chord diagram showing the functional coupling of neurons in one example session. The preferred orientation of the neurons was labeled by the lines surrounding the chord diagram. A total of 108 functional coupled pairs were detected, as indicated by the thin lines connecting neurons. e) Proportion of FC in relation to differences in preferred orientation. f) Example neuron showing consistent waveforms (above) and preferred orientation tuning (below) for more than 50 days. Averaged (in black) and 50 randomly selected trances (in gray) are shown at the top. Error bars, SEM.

To demonstrate the effectiveness of MERF in studying spiking dynamics of a large population of neurons, we characterized the coherent firing patterns of neurons in V1. One important measure to reveal the dynamic interaction among simultaneously recorded neurons is functional coupling (FC), representing sequential coactivation at millisecond‐time scale in neuronal pairs that could be related to synaptic connectivity between the cells.^[^ ^48^, ^49^ ^]^ Previous studies have characterized visual‐related FC within visual cortical areas.^[^ ^50^, ^51^ ^]^ We computed the pairwise cross‐correlations between the spiking times of each pair of MERF‐recorded SUs and identified 108 functionally coupled neuron pairs among 848 pairwise correlations examined, based on the biased spiking cross‐correlogram with peaks within the 10 ms window (**Figure** **6**a–c). Due to MERF's ability of simultaneous recording from hundreds of neurons, we typically observed more than 100 functionally coupled neurons pairs in each recording session (Figure 6d). Interestingly, we found that the neurons with similar tuning preferences were more likely to be functionally coupled (Figure 6e), in line with the previous finding.^[^ ^50^ ^]^ These results highlight the ability of MERF electrode arrays to resolve the functional organization within a local brain area.

The stability of recording SUs from the brain is critical for understanding neural circuit mechanisms underlying brain functions and for translational applications such as BMI. To evaluate the ability of MERF array in tracking individual neuronal activity, we recorded V1 neural activity evoked by same visual stimuli at regular intervals for 52 days and identified SUs putatively tracked across sessions based on their waveforms (Experimental Section).^[^ ^17^ ^]^ As shown in Figure 6f, a representative SU at a single MERF recording channel displayed similar spike waveforms and stable tuning curves across multiple recording sessions. More examples of tracked SUs at multiple channels along the same MERF array shank are shown in Figure S5 (Supporting Information). These results demonstrate the capability of MERF in longitudinally tracking the activity of neurons in the brain.

## 3 Discussion

In this study, we developed an ultrathin, mechanically robust electrode array (MERF) for SU recording from NHPs. This MERF electrode array causes minimal tissue damages (Figure 1f) and enables long‐term stable recordings in macaque cortex for up to eight months. We also demonstrated the capability of MERF in stable high‐quality SU recordings by measuring the orientation tuning and the RF of V1 neurons over a large cortical volume, and tracked individual SU activity of these V1 neurons. In addition, we showed that MERF‐recorded signals from the monkey's M1 can be decoded for the on‐line control of a computer cursor. The large number of SUs recorded by MERF enables us to reveal the high functional coupling among V1 neurons of similar orientation preferences over a large cortical area. Together, these results demonstrate that MERF provides new advances in large‐scale recordings of neural activity in the NHP brain with high spatial and temporal resolution. Furthermore, it is also feasible to simultaneously implant many MERF arrays in multiple regions in order to achieve recording at the whole‐brain level, with minimal tissue displacement. To our knowledge, only one previous study has reported long‐term recording of SUs in the cortex of NHPs using penetrating flexible probes.^[^ ^38^ ^]^ A comparison with this study highlights the advantages of our MERF arrays in terms of recording performance. First, the number of implanted electrodes in a single monkey was limited to 125 channels in that study, whereas we successfully implanted 663 channels using MERF arrays in one monkey, providing a significantly larger recording capacity. Second, for electrodes with comparable size (30 µm), they reported spiking activity on only 4% of all channels, which is substantially lower than the fraction in our results (34.6% ± 18.4%, mean ± SEM, Figure 2g). Third, our MERF array (1 µm) is significantly thinner than their probe (20 µm), which helps to reduce tissue displacement after implantation. Furthermore, MERF arrays have been implemented in chronic recording during behavioral experiments in three different laboratories, suggesting that our MERF array is a readily applicable tool for studying neural dynamics in NHPs.

Compared with the performance of “Utah” arrays reported in studies by Black et al. (first 2 weeks: 52.8%, week 24: 13.4%) and Joshi‐Imre et al. (first 2 weeks: 94.6%, week 30: 16.4%), we noted that our MERF arrays did not exhibit better performance, which can potentially be explained by the location of recording sites. In Black et al. and Joshi‐Imre et al., the recording sites of “Utah” arrays (1 mm shank length) were concentrated in layer 5 of rat motor cortex,^[^ ^52^ ^]^ where large neuronal somata densely resides and generate high‐amplitude spikes, whereases the recording sites of each MERF array were evenly distributed in a range of 2 mm below brain surface, spanning from layer 1 to layer 6 in monkey V1 and M1.^[^ ^53^, ^54^, ^55^, ^56^ ^]^ The distribution of neuronal somata is not uniform across layers and recording sites in layer 1 are unlikely to resolve spikes due to the lack of neuronal somata in this layer. When we focused on the recording performance of MERF arrays also in the layer 5 (0.865–1.497 mm) of motor cortex^[^ ^55^ ^]^ around the similar time point post implantation (week 23 to week 34), the fraction of active channels of MERF arrays varied between 20.5% and 35.9%, showing better performance than “Utah” arrays (week 24: 13.4%, week 30: 16.4%). Meanwhile, we would like to bring it up that although the linear distribution of recording sites of our MERF arrays along cortical depth may sacrifice SU yields, it can simultaneously resolve information encoded cross layers, providing a 3D spatial coverage.

MERF arrays possesses several advantages for recording neural activity in primates compared to conventional electrode arrays, such as “Utah” arrays and “Neuropixels” probes. First, the flexible nature of MERF introduced minimal tissue displacement and immune reaction. Second, due to the small size and the high channel count per shank, only a small craniotomy is required to implant a large number of electrodes. As we have demonstrated here, the M1 signals recorded from a single MERF array could support the on‐line control of the cursor movement (Figure 5), offering an effective approach for BMIs. Third, the MERF array can be customized in a variety of configurations depending on the needs of specific experiments. For example, a more compact layout of recording sites can be achieved with electron beam lithography for the dense sampling of local neural activity. In this line of previous study using electron beam lithography, a minimum linewidth of 200 nm (pitch of 400 nm) for the interconnect traces and sub‐100 nm interlayer alignment were achieved, and an electrode density of ≈3200 electrodes mm^−2^ could possibly been patterned (compared to ≈1900 electrodes mm^−2^ in Neuropixels 2.0).^[^ ^39^, ^57^ ^]^ Furthermore, the modularized implantation methods allows the scalability of the number of arrays implanted, providing flexibility in spatial coverage.

In addition, the improved mechanical strength of MERF arrays enables effective penetration into deep cortical layers and potentially subcortical regions in the monkey brain with longer shanks. Many deep brain structures play an indispensable role in different cognitive functions in our daily life, and their malfunction often causes severe neurological and psychiatric disorders.^[^ ^58^ ^]^ However, due to technical limitations, our understanding of neural dynamics of deep brain structures, especially in primates, is still superficial, leading to a lack of effective treatment strategies in clinical applications. One of the main anticipated advantages of MERF arrays in the future is the ability to examine the neural activity of deep brain structures in both health and disease at unpresented spatiotemporal resolution and coverage. Compared to the limited length of “Utah” array shanks (0.5–1.5 mm), the 2 cm long shank of MERF arrays can cover cortical layers at different depths and reach structures deeply buried in sulci. Meanwhile, compared to the silicon‐based “Neuropixels” probes whose fragile structure can only afford acute implantation into the primate brain,^[^ ^31^ ^]^ the exceptional mechanical strength and flexibility of MERF arrays enable long‐term implantation in deep brain regions. On the other hand, compared to commercial DBS (deep brain stimulation) electrodes, the high tissue compatibility and channel counts of MERF arrays may offer the possibility to achieve precise and stable modulation of neural activity at a low current of several µA,^[^ ^59^ ^]^ lowering the risk of off‐target side effects and tissue damage during stimulation.^[^ ^60^ ^]^ Therefore, we believe that the advantages of MERF arrays will facilitate revealing properties of deep brain networks and developing novel clinical therapies of more complicated neurological and psychiatric disorders, such as addiction, depression, and obsessive‐compulsive disorder.

Several challenges need to be overcome for a broader application of MERF arrays in the future. First, the longevity of stable recordings in the primate brain beyond 8 months and the feasibility of clinical applications remain to be explored. A significant extension of in vivo lifetime of the MERF array could be achieved by adding a layer of hydrophobic coating (e.g., silicon carbide) to improve its waterproof properties, and by using new adhesive materials for antidelamination. Second, we focused on the recording performance of MERF array, but the capability of MERF array for electrical stimulation remains to be demonstrated. In theory, the small dimension and the tight tissue‐electrode integration^[^ ^39^, ^61^, ^62^ ^]^ of the MERF array can be very useful in delivering spatially‐confined electric stimulation, providing a tool for modulating brain activity and investigating neural circuits in the brain. Third, we realized that electrode sites with such a high density often recorded redundant information in local neural activity in NHPs (Figure 2e), leading to increased pressure on real‐time data processing. In the future, we will optimize the spatial distribution of the electrode sites to achieve a balance between a good quality of spike sorting and an extended spatial coverage of brain tissues. Furthermore, integrating with analog switch or using multiplexer will increase signal processing efficiency by only recording user‐selected channels.

## 4 Conclusion

Understanding the dynamics of neural circuit activity underlying various brain functions requires long‐term monitoring of neuronal spiking over large populations of neurons with high spatial and temporal resolution. However, the longevity and stability of recording in NHPs remain challenging. In this work, we successfully developed a mechanically robust ultraflexible, 1 µm thin electrode array (MERF) which allows stable recording of neural activity in the monkey brain with SU resolutions over long duratons. Our results demonstrate that the MERF array possesses significant advantages over the existing techniques used for recordings in NHPs. Given the minimal invasiveness, stable functionality, and flexibility of spatial configurations, MERF arrays are well‐suited for studying neural circuit mechanism underlying cognitive functions in primates and for clinical applications such as neuroprothetics.

## 5 Experimental Section

### Design, Fabrication, and Assembly of MERF Electrode Array

Each MERF electrode array contained 128 channels for electrophysiological recording in vivo. The array consisted of two flexible implantable shanks, each containing 64 recording sites aligned at the edge of the shank at 30 µm pitch, starting from the shank tip. Each recording site was in circular shape with a diameter of 25 µm. The flexible shank was 20 mm in length. The shank spacing can be customized by adjusting the spacing between the two shuttling tungsten wires by which the flexible shanks were inserted into brain tissues. The recording sites protruded slightly from the edge of the shank for better signal quality.^[^ ^65^ ^]^

The fabrication method of MERF was adapted from a planar microfabrication technique featuring a multilayer architecture, as described in previous publications (Figure S6, Supporting Information).^[^ ^17^ ^]^ Compared to the previous design (NET probes), the MERF array incorporates several improvements. First, to achieve a higher tensile strength of the MERF arrays, the entire structural and passivation material was updated from SU‐8 to nonphotosensitive PI and patterning was achieved by O_2_ plasma etching. Second, Titanium instead of Chromium was used as the adhesion layer between metal and polymer. Third, the MERF array featured more recording sites (64 sites) per shank, whereas the NET probe shank only contained 8 recording sites. This increase in the number of recording sites allowed for more comprehensive sampling of neural activity. Last, the recording sites of the MERF array were designed to slightly protrude from the edge of the shank. This design modification was implemented to enhance signal quality.^[^ ^65^ ^]^ In contrast, the recording sites of the NET probes were positioned at the middle of the shanks. The overall device thickness was limited to 1–1.5 µm to retain the low bending stiffness of the shank. Both the interconnect and surface of the recording sites were made of gold. A hole in 20 µm diameter was designed at the tip of the flexible shank, in which the tip of the shuttling tungsten wire was anchored to drag the shank into brain tissues during implantation. The surfaces of the recording sites were sputtered with 200 nm of iridium oxide film (Kurt J. Lesker, LAB18; sputtering conditions: 10^−6^ Pa vacuum, 150 W, 20 sccm O_2_, and 50 sccm Ar) or electrochemically deposited PEDOT:PSS coating to lower the electrode impedance to 100 kΩ at 1 kHz in saline solution.

Each MERF array was soldered to a 128‐channel FPC (flexible printed circuit) board of 42 mm in length, which was connected to the Intan or SpikeGadget headstage for signal acquisition. Two shuttling tungsten wires were fixed on a carrier chip with 5% PEG‐300000. The tungsten wire was electrochemically etched to form a step with a 10 µm thin column at the tip (Figure S7, Supporting Information).^[^ ^62^ ^]^ After the implantation surgery, the monkeys carried multiple MERF arrays protected by a customized chamber (Figure S3, Supporting Information).

### Finite Element Analysis

In real‐life handling, the tip region of the MERF array shank is the weakest part to carry the load during insertion through the pia mater. In FEA, a model of the MERF tip was used to simulate the bending and stretching deformation under stress to determine the breaking load and the load required to bend the probe tip, as an indicator of the strength and flexibility of the probe, respectively. The workflow of FEA includes 3D reconstruction and meshing, material property assignment, load, and boundary condition setting, and load‐strain/deformation relationship analysis.

SolidWorks 2021 was used to construct 3D finite element models of the probe (1 and 7 µm thick, respectively), and ANSYS Workbench was used to generate a tetrahedral mesh for the 3D geometry, with an element size of 1 µm, and to perform FEA. PI and SU‐8 were the two materials tested for the MERF insulation body. The material parameters of polyimide were obtained from the *PI‐2600 Series* (HD MicroSystems, www.hdmicrosystems.com), and the material parameters of SU‐8 were sourced from *SU‐8 2000* (KayakuAM, www.kayakuAM.com). Material constants for PI and SU‐8 were registered as the following: Young's modulus of PI and SU‐8 were 8.5 and 2.0 GPa, respectively, and tensile strength of 350 and 60 MPa, fracture strain of 100% and 6.5%, Poisson's ratio of 0.34 and 0.22, respectively. For an element subjected to tensile stress, failure was defined as the initiation when the maximum principal stress reached the critical value (i.e., tensile strength). The boundary conditions are presented in Figure S8 (Supporting Information); and Figure 1d. In the stretching simulation, a uniaxial stretching load horizontal to the probe surface was applied to the distal arc surface of the tungsten wire hole with uniform load distribution. All nodes before the first recording electrode were completely constrained. The applied load was increased stepwise, starting from 0 to 100 µN for SU‐8 (1 µm), 600 µN for PI (1 µm), and 5 mN for PI (7 µm). The same constraints were used for bending simulation, but a torque around the *y*‐axis was applied to a point anterior to the tungsten wire hole (Figure S8, Supporting Information).

### Animals

All animal care, housing, and experimental procedures were approved by the Chinese Academy of Science, Institute of Neuroscience Institutional Animal Care and Use Committee, and conformed to the principles outlined in the Guide for the Care and Use of Laboratory Animals. Two female cynomolgus monkeys (*Macaca fascicularis*) and one male rhesus monkey (*Macaca mulatta*), #1, #2, and #3 (5.8, 4.9, and 8.5 kg; 14, 10, and 8 years old, respectively), were used in the experiments. One MERF array was implanted in the M1 of the left hemisphere of monkey #1. Seven and three MERF arrays were implanted into the V1 of the left hemisphere of monkey #2 and monkey #3, respectively (Table S2, Supporting Information).

### Surgical Procedures

A titanium post for head fixation was implanted 2 months before electrode implantation. Implantation surgeries were performed under full anesthesia. The monkeys were initially anesthetized with an intramuscular dose of Zoletil 50 (5 mg kg^−1^), maintained by endotracheal intubation with 3% isoflurane, and mounted on a stereotaxic apparatus. Electrocardiography (ECG) and oxygen saturation monitors were attached to the animals to monitor vital signs. After the scalp sterilization procedures, a scalp incision of 4 cm was made at the occiput area, exposing the skull beneath. Six to eight skull screws were placed in the drilled craniotomies, on which the customized chamber base ring was fixed using light‐curing dental acrylic (3M).

Inside the chamber, a cranial window of 5 × 3 mm was created using a dental drill. The dura mater was then incised to expose the underlying cortex. The MERF arrays were implanted one by one in a modularized manner. A MERF array was first attached to a customized vacuum holder mounted on a micromanipulator arm, then propelled into the target brain area with a solenoid, with an implant depth of ≈2 mm. The 2 mm insertion depth was chosen based on the average thickness of the monkey cortex^[^ ^66^ ^]^ and MRI data of monkeys involved in this study. The push‐pull solenoid provided a fixed traveling distance (*D1*). To achieve a precise control of implantation depth, the micromanipulator was utilized to adjust the distance between the brain surface and the electrode tip (*D2*) before implantation. The depth of implantation was equal to *D1* minus *D2*. Following the insertion of shuttle wires, all the electrode sites were meticulously examined under a microscope to confirm their successful implantation into the cortex, with the top site just beneath the cortical surface. The tungsten shuttle wires were then retracted manually. After removing the shuttle wires, all electrode sites were examined again under a microscope to verify that they all remained in place and were not pulled out of brain tissue. The silicon carrier chip was fixed on the skull surface using dental acrylic (BRILDENT). After the adhesive was set, the first electrode array was detached from the manipulator to load the second electrode array onto the vacuum holder. The same procedure was repeated until all electrode arrays were implanted (5–10 min per 128‐channel electrode module).

After implantation, the cranial window was sealed with the human fibrin sealant (FIBINGLUEAAS, Shanghai). A subsequent layer of dental acrylic was applied over the craniotomy and surrounding skull surface to firmly fix the silicon carrier chips to the skull and chamber. Finally, a customized cap was placed to cover the chamber and secured using four screws.

### Visual Stimulus

The monkey #2 and monkey #3 were trained to perform the fixation tasks. An LCD screen (ZHIIXANDA, 21′, 1920 × 1080) was used to present the visual stimuli. An eye tracker (Eyelink 1000 +) was used to track the fixation point of the animal. The distance between the monkey's eye and the screen was 74 cm. Visual stimuli were generated using the OPTICKA toolbox (https://github.com/iandol/opticka), which is based on Psychtoolbox‐3 (http://psychtoolbox.org/). A sine wave grating with a contrast of 0.5 was used to study the neuron's orientation selectivity. The RFs of the neurons were mapped with a grating disk (1^o^ visual angle) randomly presented at different locations on the screen.

### Center‐Out Task

The monkey #1 was trained to manipulate a joystick with the hand contralateral to the implantation hemisphere to move a cursor to multiple targets. The cursor and targets were presented on an LCD monitor with a refresh rate of 60 Hz controlled by a computer using Matlab 2021b. Joystick signal was measured at 20 Hz with an Arduino UNO connected to the PC. The cursor shape was designed as a 2.5 cm diameter blue circle which was located in the center of the screen at the beginning of each trial. The target was one of the eight red circles (3.7 cm in diameter, 7.5 cm away from the center) peripherally located around the center and alternated randomly on the screen across trials (8 directions in total, 45^°^ between two adjacent targets). A decoder based on the preferential subspace identification algorithm^[^ ^67^ ^]^ was initialized using the cursor and neural spiking activity during hand control. The neural spiking activity was generated by applying high‐pass filter (>300 Hz, 4th‐order Butterworth filter) to notch‐filtered (50 Hz) broadband signals and then crossing threshold at −70 µV. In brain control sessions, the monkey still had access to the joystick but the joystick was unplugged to the system. The decoder was first calibrated and then used simultaneously recorded neural spiking activity to update the cursor's 2D velocity. The accuracy, time cost, and path efficiency were measured to evaluate the task performance. Time cost was defined as the time of the center cursor reaching the surrounding targets. Path efficiency was defined as the ratio between the shortest path distance to reach the surrounding target and the actual path distance traveled by the cursor.

### Electrophysiological Recording and Spike Sorting

In most cases, broadband signals were recorded at 20 kHz using the Intan RHD2000 evaluation system, along with impedance measurements before and/or after electrophysiological recording. Where visual stimuli needed to be presented to the monkeys, SpikeGadgets modular headstages were used for electrophysiological recording, so that all electrodes were simultaneously recorded during the visual stimulus presentation. Synchronization between visual stimulation events and electrophysiological recordings was achieved by sending TTL signals from the visual stimuli presenter, OPTICKA, through a LabJack U3 to the SpikeGadgets main control unit. As for M1, electrophysiological signals were recorded at 30 kHz using the Intan RHD2000 evaluation system, along with impedance measurements before electrophysiological recording. Raw signals recorded from each channel were first band‐pass filtered by an on‐chip DSP with a 0.1 Hz to 7.5 kHz passband filter and then filtered with a digital notch filter at 50 Hz, followed by a band‐pass filter from 250 Hz to 7.5 kHz. Synchronization between the joystick signals, cursor on the LCD screen, and electrophysiological recordings was achieved by sending TTL signals from the behavioral task computer to the Intan RHD2000 digital input channels using the Arduino UNO.

Raw signals were low pass filtered (0.3–300 Hz) to obtain the LFP. Spike sorting was performed using Kilosort 3 (https://github.com/MouseLand/Kilosort) for recording from M1 of monkey #1, and MountainSort v4 (https://github.com/magland/ml_ms4alg) for recording from the V1 of monkey #2 and monkey #3. Broadband signals were first applied with common median referencing from each electrode (to reduce the common‐mode noise, such as motion artifacts), high‐pass filtered (500–5000 Hz), high‐voltage artifacts removed (by replacing chunks of data with amplitudes outside the range of six standard deviations by zeros in a 0.1 s moving window), and whitening filtered. The preprocessed signals were subjected to spike detection at a threshold of 4.5 standard deviations of the background signal in both positive and negative directions. An adjacency radius of 100 µm was applied. Data points of 1.6 ms were saved for each spike (0.4 ms before and 1.2 ms after the peak/trough). For data from monkey #1, manual selection was performed with Phy (https://github.com/cortex‐lab/phy/). Drift correction was off due to relatively short recording duration (median: 30 min) and head‐restriction. For data from monkey #2 and monkey #3, a curation process (adopted from Mountainsort v3^[^ ^41^ ^]^) was performed to accept clusters of spikes using the following criteria: firing rate >0.05 Hz, isolation threshold >0.95, noise overlap threshold <0.03, and peak SNR>1.5. The outlier waveforms were further removed from these curated clusters using the generalized extreme studentized deviation test for outliers. Clusters whose fraction of spikes with inter‐spike intervals within 1.6 ms was higher than 2% of the total firing events were discarded. Pools of putative SUs were finalized by merging clusters around the adjacency radius, as mentioned above, by consistency in their spiking events.

To identify putative same SUs tracked across recording sessions, principal component (PC) analysis of spike waveforms was performed and the distance between the cluster centers from two adjacent recording sessions was examined, as previously described in the same line of work.^[^ ^17^ ^]^ If the distance between the cluster centers was less than 2σ of the PC distribution in the earlier session of the session pair, the waveforms from these two sessions were considered from the same SU. In the example of Figure 6f, the distance between the cluster centers from two adjacent recording sessions was less than 0.95σ, suggesting that the same SU was tracked across recording sessions putatively.

### Preferred Direction of Hand Movements

A vector for each SU in M1 was calculated to represent its preferred direction of hand movements (Figure 5d). The projections on *x* and *y* axes of the mean firing rate (*FR_i_*) (0–300 ms relative to the movement onset) summed from 8 directions (θ_*i*_) were calculated as

(1) $$FR_{x}=\frac{\sum_{i=1}^{8}FR_{i}\cos\theta_{i}}{\max\left(FR_{i}\right)}$$

(2) $$FR_{y}=\frac{\sum_{i=1}^{8}FR_{i}\sin\theta_{i}}{\max\left(FR_{i}\right)}$$

Then, the preferred direction and preference strength were calculated by the degree and the length of vector [*FR_x_*, *FR_y_*].

### Functional Coupling Analysis

FC of the neurons was detected using baseline‐corrected cross‐correlation, as previously reported.^[^ ^68^ ^]^ Briefly, cross‐correlations (0.4 ms binning) were generated with the spike trains from each neuron ranging at lags from −200 to 200 ms. The observed CCG (cross‐correlogram) was convolved with a partially hollow (60%) Gaussian kernel with a standard deviation of 10 ms to generate the lower frequency baseline, from which the peak in the CCG must exceed for acceptance of the presence of synchrony between neurons. Only the FC in the causal direction was considered, where the peak in the positive lag was significantly larger than that in the negative lag.

### Histology and Imaging

One cynomolgus monkey died because of natural causes. After the animal death, the cadaver was perfused transcardially with 10% formalin (Sinopharm) at a rate of 500 mL h^−1^ until the exudate was clear. The brain tissue containing implanted electrodes was carefully extracted, placed in fresh 10% formalin, and stored at 4 °C for 48 h. Before sectioning, brain tissue was cryoprotected by equilibration in a stepwise gradient of sucrose (10–20–30%, Sigma) in 1 × PBS at 4 °C. After equilibration, tissue was frozen at −25 °C in O.C.T. compound (Tissue‐Tek, 4583), sliced horizontally into 20 µm sections, and mounted onto slides to be stored at −80 °C until immunohistochemical labeling.

The sections were immunohistochemically stained using a standard immunohistochemical protocol. Briefly, the tissue slices were washed three times with 1 × PBS, and the cell membranes were permeabilized by 30 min incubation in 1 × PBS containing 0.5% Triton‐X 100 (Sigma, 1X PBS‐T). The slices were blocked with 3% BSA in 0.1% PBS‐T for 1 h at room temperature before incubation with primary antibody (overnight at 4 °C). The primary antibodies used were rat antiglial fibrillary acidic protein (GFAP, 1:400, #13‐0300, Invitrogen) and guinea pig antineuronal nuclei (NeuN, 1:2000, #ABN90, Sigma‐Aldrich) diluted in 0.1% PBS‐T. The extra primary antibodies were removed by three 1 × PBS washes, and the appropriate Alexa Fluor conjugated secondary antibodies (diluted at 1:1000 in PBS buffer) were added to the brain sections and incubated at room temperature for 2 h. After three subsequent 1 × PBS washes, the slices were mounted with DAPI Fluoromount‐G (Southern Biotech, 0100‐20) for imaging. Fluorescent images were acquired using a Nikon A1 confocal laser microscope with a 20 × objective.

### Statistical Analyses

Sample sizes were predetermined without any statistical methods but based on those generally employed in the field. Statistical significance was defined by alpha preset to 0.05. Error bars indicate standard errors of the mean (SEM) unless noted otherwise. Formular for mixed‐effects model in Figure 4e is as below

(3) $$\begin{array}{ccc} \mathrm{RF}\;\mathrm{size}\;\approx \;1 & + & \mathrm{recording}\;\mathrm{site}+\left(1|\mathrm{shank}\right)\\ & & +(\mathrm{recording}\;\mathrm{site}\;-1|\mathrm{shank}) \end{array}$$

where (1|shank) and (recording site  − 1|shank) indicate a random effect constant and a random effect slope term for each shank, the coefficient of recording site was tested against 0.

### Ethical Statement

All animal care, housing, and experimental procedures were approved by the Chinese Academy of Science, Institute of Neuroscience Institutional Animal Care and Use Committee, and conformed to the principles outlined in the Guide for the Care and Use of Laboratory Animals.

## Conflict of Interest

The authors declare no conflict of interest.

## Author Contributions

Y.T., J.Y., and C.W. contributed equally to this work. Z.Z., X.L., and C.L. conceived and organized the overall study. Z.Z., C.L., T.Y., Y.T., and J.Y. designed the experiments with inputs from all authors. Z.Z., X.L., and C.W. designed and fabricated the ultraflexible electrodes. Y.T. and J.X. assembled and prepared devices for surgeries. J.Y. and Z.H. designed the protective chambers. Z.Z., Y.T., and J.Y. developed, optimized, and performed surgical procedures. J.Y., Z.H., Y.X., X.F., Y.Z., T.M., T.Y., and Y.T. performed animal behavioral experiments, neural recording, and data analysis supervised by C.R., C.L., T.Y., and Z.Z. J.X., Y.T., and J.Y. performed histology supervised by Z.Z.. Y.T., C.R., J.Y., J.X., and Z.H. wrote the manuscript with inputs and revisions from all authors.

## Supporting information

Supporting Information

Supplemental Movie 1

Supplemental Movie 2

Supplemental Movie 3

## Data Availability

The data that support the findings of this study are available from the corresponding author upon reasonable request.
