# Supplementary material for: An Ultraflexible Electrode Array for Large‐Scale Chronic Recording in the Nonhuman Primate Brain
Source: Adv Sci (Weinh). 2023 Oct 23;10(33):2302333. doi: 10.1002/advs.202302333 (PMC10667845; doi:10.1002/advs.202302333)
Supplement: Supplementary file 1 — Supporting Information [file ADVS-10-2302333-s002.pdf]

ADVANCED SCIENCE
Open Access

Supporting Information

for *Adv. Sci.*, DOI 10.1002/advs.202302333

An Ultraflexible Electrode Array for Large-Scale Chronic Recording in the Nonhuman Primate Brain

Yixin Tian, Jiapeng Yin, Chengyao Wang, Zhenliang He, Jingyi Xie, Xiaoshan Feng, Yang Zhou, Tianyu Ma, Yang Xie, Xue Li, Tianming Yang, Chi Ren, Chengyu Li* and Zhengtuo Zhao**

Supporting Information

An ultra-flexible electrode array for large-scale chronic recording in the non-human primate brain

Yixin Tian, Jiapeng Yin, Chengyao Wang, Zhenliang He, Jingyi Xie, Xiaoshan Feng, Yang Zhou, Tianyu Ma, Yang Xie, Xue Li, Tianming Yang, Chi Ren, Chengyu Li*, Zhengtuo Zhao**

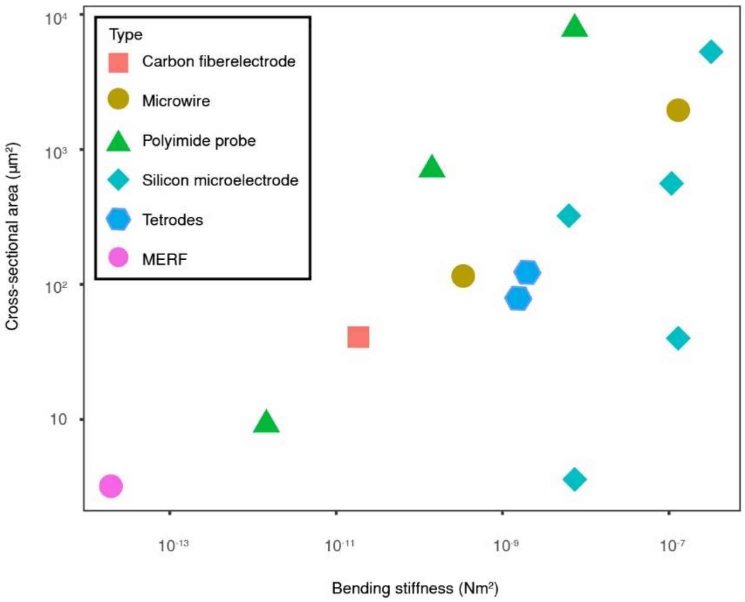

Figure S1. Comparison of previously reported neural electrodes with MERF in the cross-sectional area per electrode and bending stiffness. All recordings made in primates.

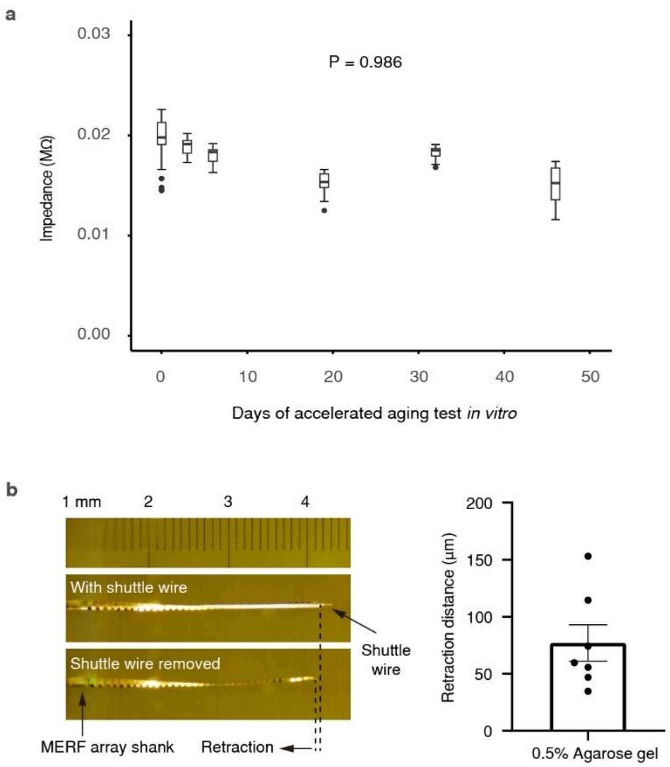

Figure S2. *In vitro* test of MERF arrays. a) The impedance of the MERF array remained stable throughout the 46-day accelerated aging test (maintained at a temperature of 60 ℃ in 1× PBS, n = 31 electrode sites, p = 0.986, ANOVA test). Boxed plot: black lines indicate median, bottom and top box edges indicate percentiles of 25% and 75%, respectively. b) In vitro tests of insertion depth accuracy. Left, images showing the same MERF array shank before and after removing the shuttle wire in 0.5% agarose gel. Right, retraction distance (n = 7 shanks, mean ± SEM).

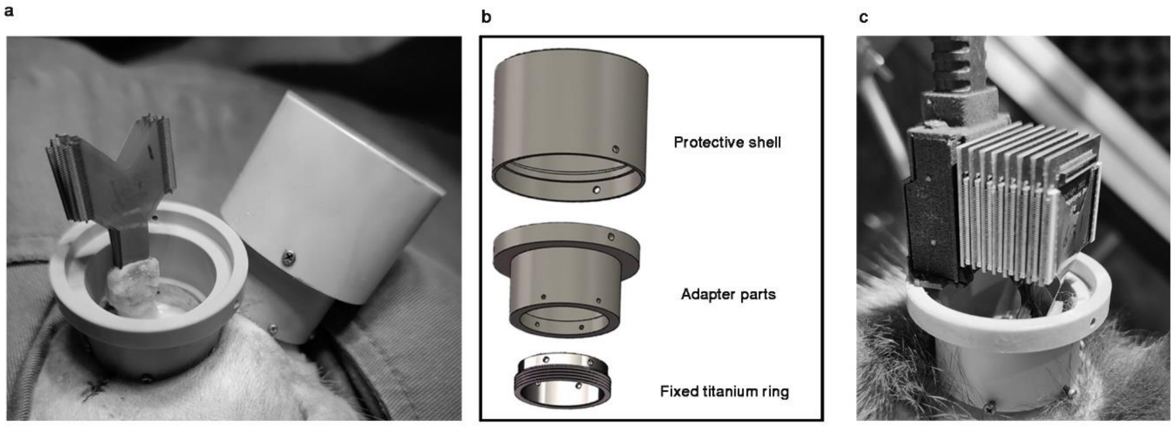

Figure S3. Implantation of multiple MERFs in the primate brain. a) Stacked MERF electrode arrays protected by a customized chamber. b) Diagram showing individual components of the customized chamber. c) Stacked headstages enabled simultaneous recording of 896 channels in a *Macaca fascicularis* monkey.

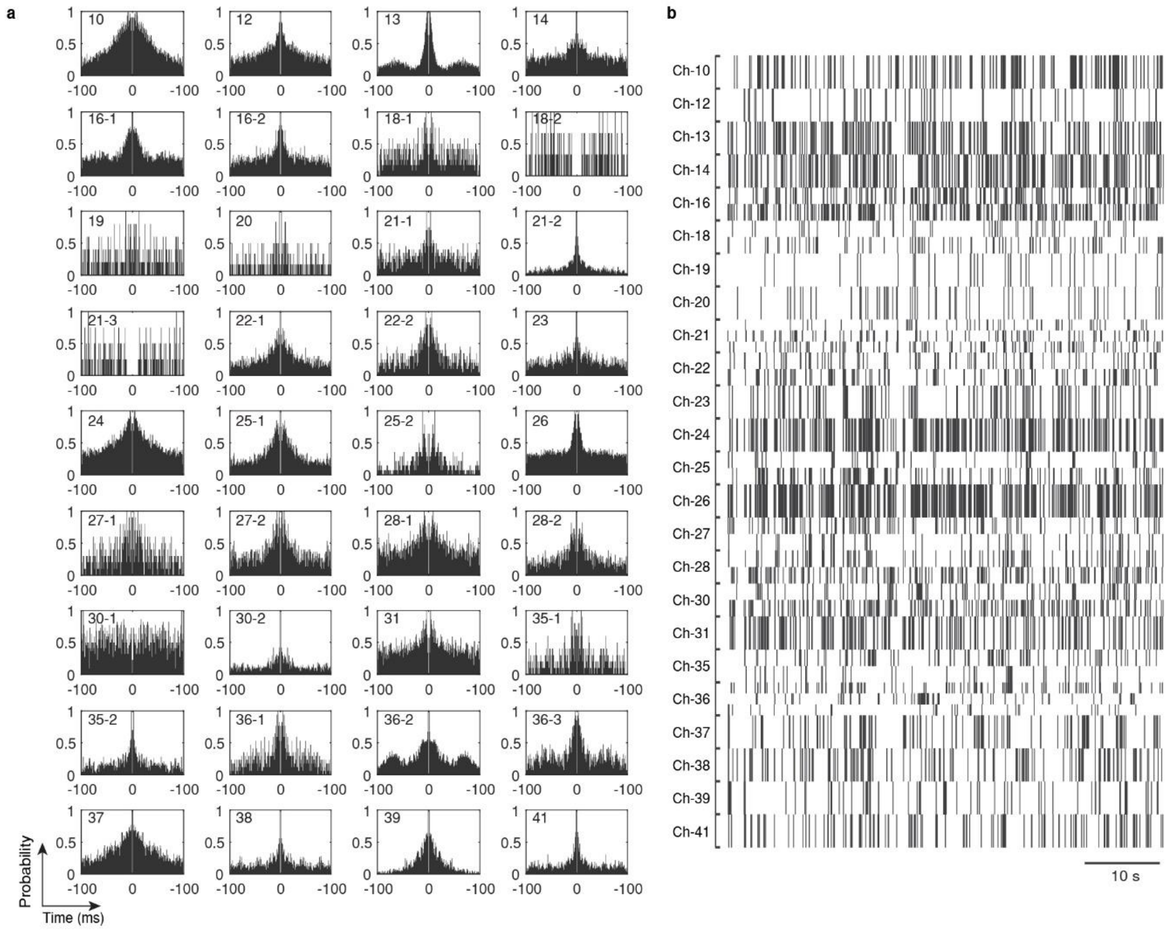

Figure S4. The SU firing properties recorded from the monkey #2 at 53 days post implantation. a) Normalized auto-correlograms of SU spikes in Figure 2b. The number in the upper-left corner of each panel indicate the channel index (1 to 64, shank top to bottom), with the neuron number following the dash (in channels with more than one neuron recorded). b) Raster plots of 1-min snippets of the SU firing shown in Figure 2b, arranged with the same channel order as in (a).

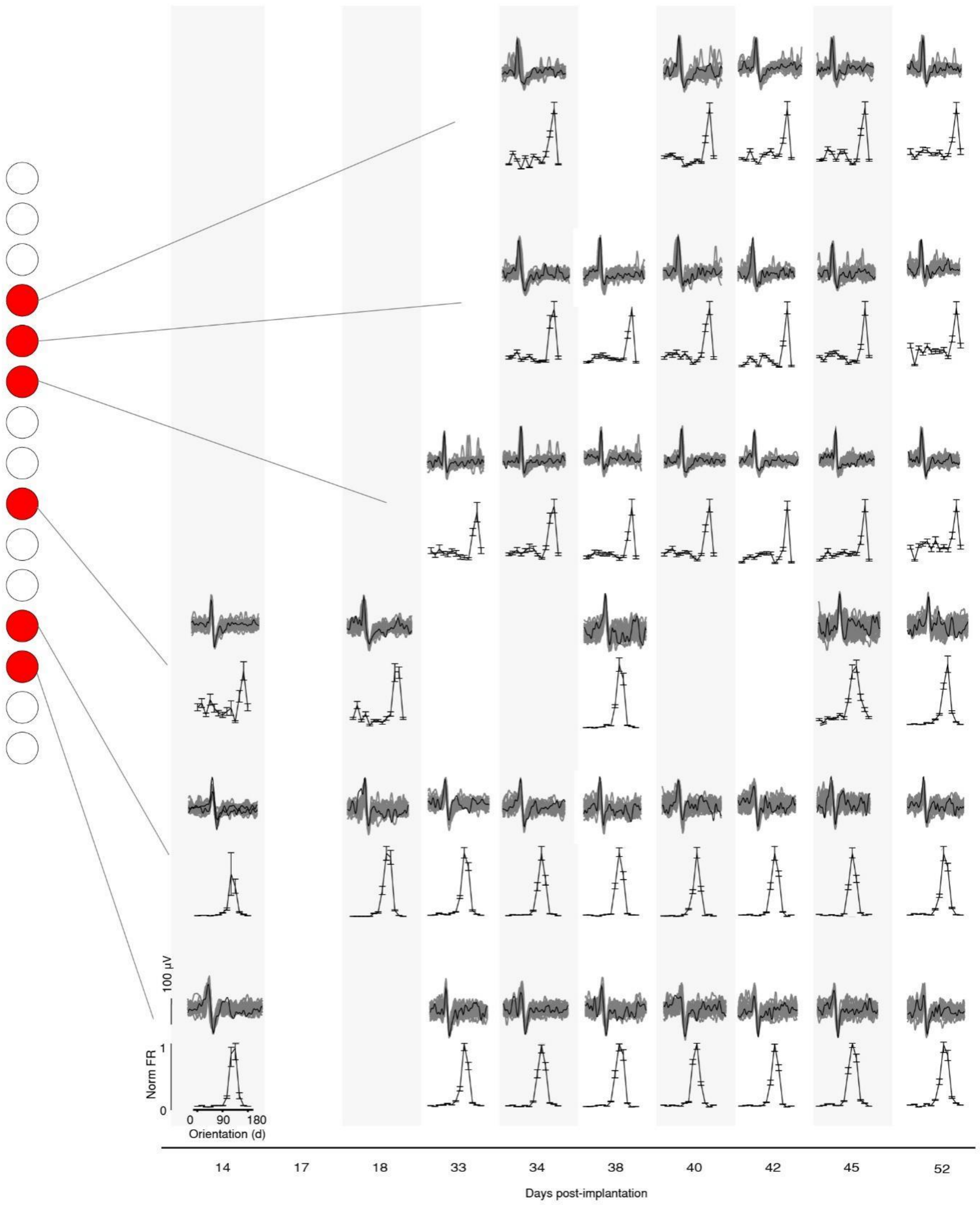

Figure S5. The waveforms and orientation tuning of SUs that were tracked over prolonged periods in V1 in monkey #2. Upper traces, SU spike waveforms (solid line, average of x traces); lower curves, tuning responses of each SUs for the orientation of visual stimuli (drifting

gratings). Recoding sites along the MERF electrode array are marked by red color. Error bars, SEM; FR: firing rate.

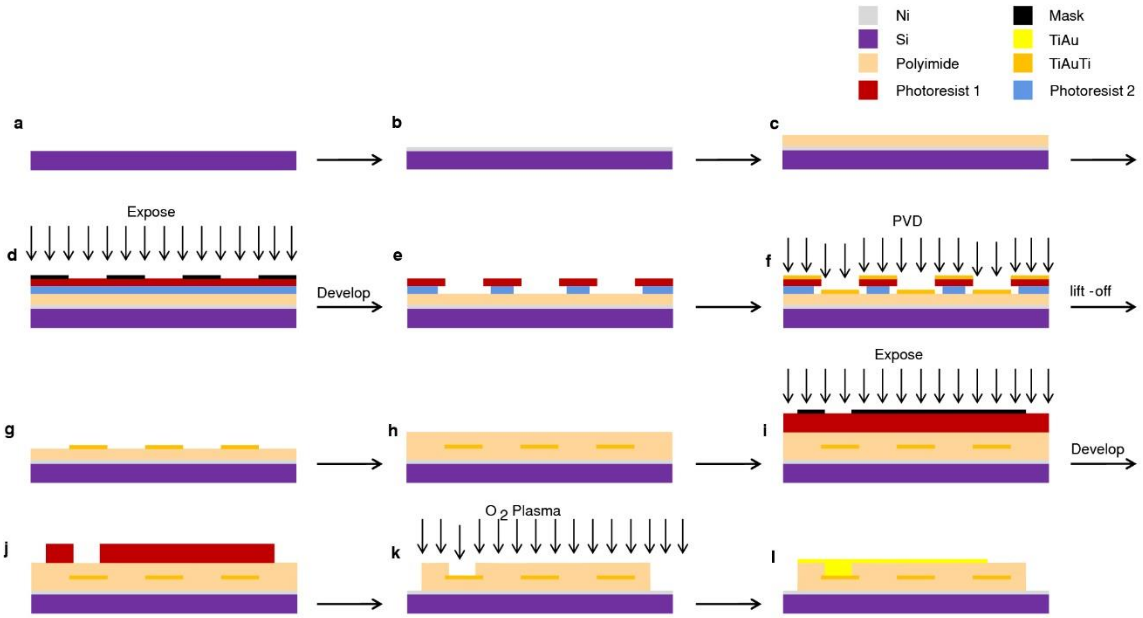

Figure S6. The flow chart for the fabrication of MERF electrode array. a) Clean Si wafer with a 300-nm SiO_2 layer. b) Ni layer was deposited via e-beam evaporation. c) The bottom PI layer was spin-coated and cured. d) Exposure process. A double-layer resist method was applied. e) Photoresist developed by the developer. f) Au layer was deposited. g) The lift-off process for obtaining the pattern of interconnects. h) The top PI layer was spin-coated and cured. i) A thick layer of photoresist was applied to protect the PI. j) The development for photo-masked pattern. k) O_2 plasma etching for obtaining PI pattern. l) Fabrication of an Au layer for the electrode site, using the same procedure as in (d)-(g).

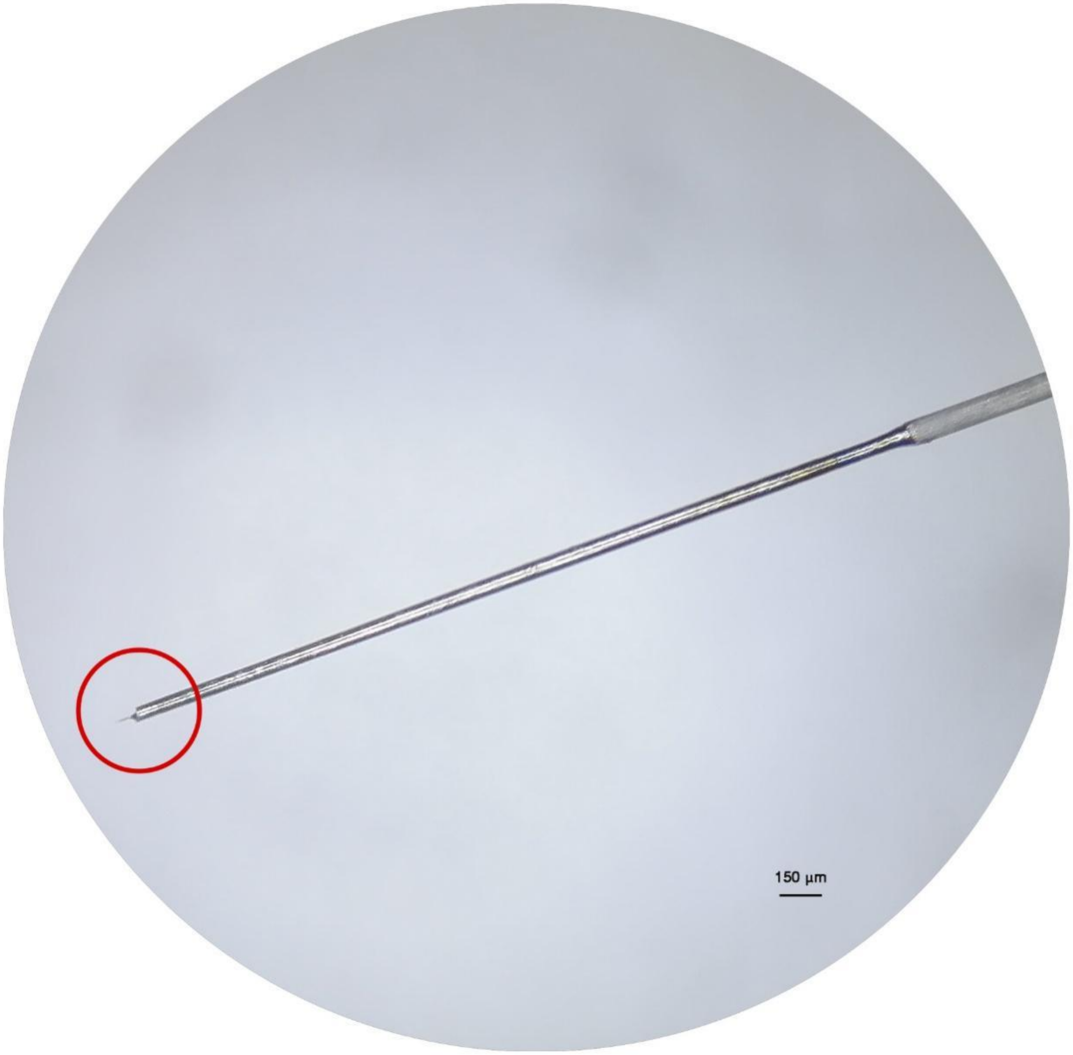

Figure S7. Microscopic image showing a tungsten wire that was electrochemically etched to form a sharp tip. Red circle, the tip of the tungsten wire.

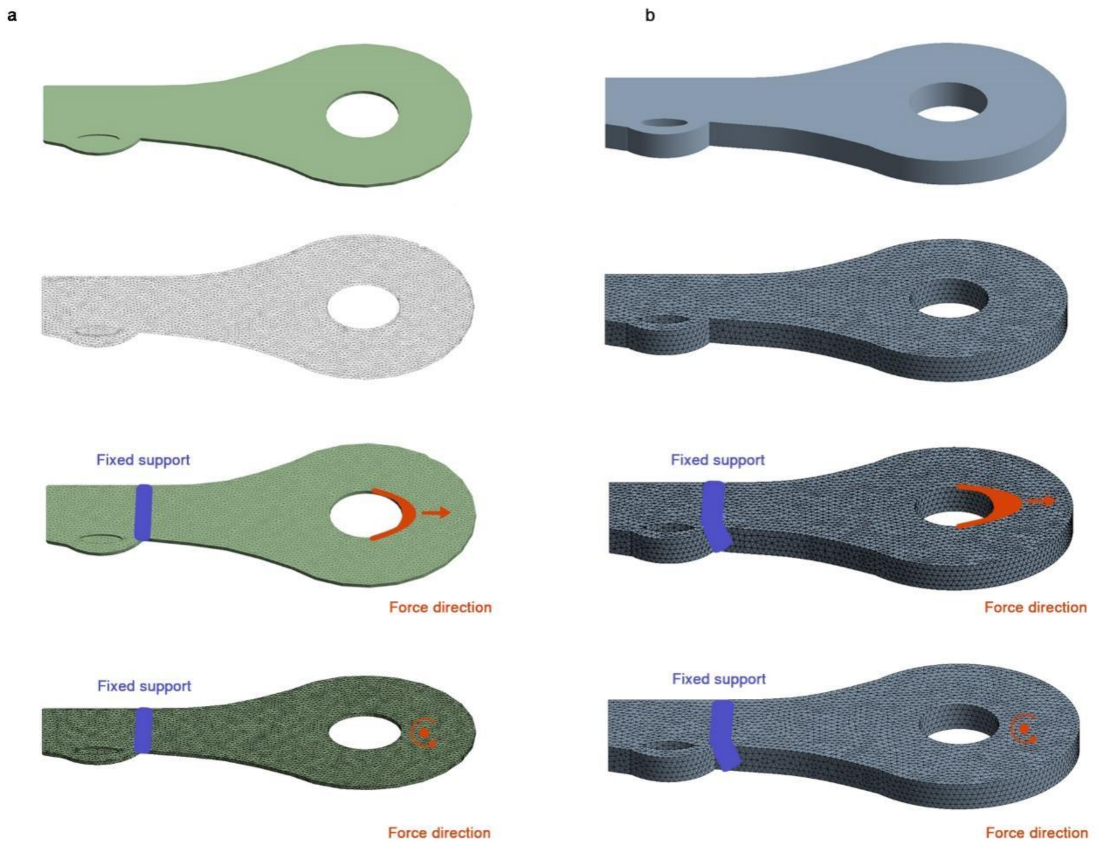

Figure S8. Schematic diagram for the 3D configuration, finite element meshing, and boundary conditions of the MERF with 1-μm (a) and 7-μm (b) thickness. Elements for the load condition marked by colors: purple, fixed support; red, force application site; red arrow, direction of force application.

Table S1. Representative neural probes in primate implantation. (d: diameter, t: thickness, w: width)

Neural Probes Type		Shank Dimensions [μm]	Surgical Footprint [μm²]	Total # of Electrodes	Cross-sectional area per channel [μm²]	References
Silicon microelectrode	Utah array	80 (d)	502,400	96	5,233.33	M. Velliste [1]
		80 (d)	502,400	96	5,233.33	X. Sun [2]
		80 (d)	502,400	96	5,233.33	H. Ma [3]
	Neuropixels	20 × 70 (t,w)	1,400	384	3.65	E. M. Trautmann [4]
		20 × 70 (t,w)	1,400	384	3.65	Q. F. Wang [5]
	Other	10 × 500 (t,w)	5,000	16	312.5	F. Barz [6]
		50 × 100 (t,w)	5,000	128	39.1	L. Klein [7]
		30 × 300 (t,w)	9,000	16	562.5	S. Zhang [8]
Microwire		50 (d)	1,960	1	1,960	S. Debnath [9]
		12 (d)	113	1	113	D. B. McMahon [10]
Tetrodes		17-25 (d)	227-491	4	56.75-122.75	L. Santos [11]
		20 (d)	314	4	78.5	D. Aronov [12]
Carbon fiber electrode		7 (d)	38	1	38.47	E. W. Schluter [13]
Parylene		20 × 1,400 (t,w)	NA (ECoG)	32	875	T. Kaiju [14]

**Polyimide probe**	24 × 2,500 (t,w)	502,400	64	7,850	F. Pothof [15]
	20 × 100 (t,w)	2,000	3	666.7	L. Merken [16]
	5 × 50 (t,w)	1,256[a)]	32	7.81	E. Musk [17]
	1 × 100 (t,w)	1,963[a)]	64	1.56	This Work

[a)]including insertion shuttles

Table S2. Summary of animals used in current study.

Laboratory	Animal #	Subspecies	Basic information	Implanted electrodes	Implantation area	Recording duration
Lab 1	1	*Macaca fascicularis*	Female 14 years old	1 array	M1	8 months
Lab 2	2	*Macaca fascicularis*	Female 10 years old	7 arrays	V1	2 months
Lab 3	3	*Macaca mulatta*	Male 8 years old	3 arrays	V1	4 months

Movie S1. A MERF array shank disturbed by a thin tungsten wire in water solution, demonstrating the high flexibility of the shank.

Movie S2. Receptive fields of simultaneously recorded SUs at different depth of the V1, reconstructed in 3D space.

Movie S3. On-line control of cursor movement using M1 neural signals recorded by MERF. Note that the joystick was disconnected from the system.

References

1. Velliste, M., et al., *Cortical control of a prosthetic arm for self-feeding.* Nature, 2008. **453**(7198): p. 1098-101.
2. Sun, X., et al., *Cortical preparatory activity indexes learned motor memories.* Nature, 2022. **602**(7896): p. 274-279.
3. Ma, H., et al., *Processing of motion boundary orientation in macaque V2.* Elife, 2021. **10**.
4. Trautmann, E.M., et al., *Accurate Estimation of Neural Population Dynamics without Spike Sorting.* Neuron, 2019. **103**(2): p. 292-308.e4.
5. Wang, Q. and H. Cui, *Reinforcement of Neuropixels probes for high-density neural recording in non-human primates*. 2021. 128-131.
6. Barz, F., et al., *Versatile, modular 3D microelectrode arrays for neuronal ensemble recordings: from design to fabrication, assembly, and functional validation in non-human primates.* J Neural Eng, 2017. **14**(3): p. 036010.
7. Klein, L., et al., *High-density electrophysiological recordings in macaque using a chronically implanted 128-channel passive silicon probe.* J Neural Eng, 2020. **17**(2): p. 026036.
8. Zhang, S., et al., *A silicon based implantable microelectrode array for electrophysiological and dopamine recording from cortex to striatum in the non-human primate brain.* Biosens Bioelectron, 2016. **85**: p. 53-61.
9. Debnath, S., et al., *Long-term stability of neural signals from microwire arrays implanted in common marmoset motor cortex and striatum.* Biomed Phys Eng Express, 2018. **4**(5).
10. McMahon, D.B., et al., *One month in the life of a neuron: longitudinal single-unit electrophysiology in the monkey visual system.* J Neurophysiol, 2014. **112**(7): p. 1748-62.
11. Santos, L., et al., *A novel tetrode microdrive for simultaneous multi-neuron recording from different regions of primate brain.* J Neurosci Methods, 2012. **205**(2): p. 368-74.
12. Aronov, D., et al., *Neural coding of spatial phase in V1 of the macaque monkey.* J Neurophysiol, 2003. **89**(6): p. 3304-27.
13. Schluter, E.W., et al., *Real-time dopamine measurement in awake monkeys.* PLoS One, 2014. **9**(6): p. e98692.
14. Kaiju, T., et al., *High Spatiotemporal Resolution ECoG Recording of Somatosensory Evoked Potentials with Flexible Micro-Electrode Arrays.* Front Neural Circuits, 2017. **11**: p. 20.
15. Pothof, F., et al., *Chronic neural probe for simultaneous recording of single-unit, multi-unit, and local field potential activity from multiple brain sites.* J Neural Eng, 2016. **13**(4): p. 046006.
16. Merken, L., et al., *Thin flexible arrays for long-term multi-electrode recordings in macaque primary visual cortex.* J Neural Eng, 2022. **19**(6).
17. Musk, E., *An Integrated Brain-Machine Interface Platform With Thousands of Channels.* J Med Internet Res, 2019. **21**(10): p. e16194.
